# Oxidative stress in androgenetic alopecia

**Published:** 2016

**Authors:** BE Prie, L Iosif, I Tivig, I Stoian, C Giurcaneanu

**Affiliations:** * ”Carol Davila” University of Medicine and Pharmacy, Bucharest, Romania; ** “Elias” University Emergency Hospital, Bucharest, Romania; *** R&D IristLabmed, Bucharest, Romania

**Keywords:** androgenetic alopecia, oxidative stress, antioxidants, erythrocytes, trolox equivalent antioxidant capacity

## Abstract

**Rationale:**Androgenetic alopecia is not considered a life threatening disease but can have serious impacts on the patient’s psychosocial life. Genetic, hormonal, and environmental factors are considered responsible for the presence of androgenetic alopecia. Recent literature reports have proved the presence of inflammation and also of oxidative stress at the level of dermal papilla cells of patients with androgenetic alopecia

**Objective:**We have considered of interest to measure the oxidative stress parameters in the blood of patients with androgenetic alopecia

**Methods and results:**27 patients with androgenetic alopecia and 25 age-matched controls were enrolled in the study. Trolox Equivalent Antioxidant Capacity (TEAC), malondialdehyde (MDA) and total thiols levels were measured on plasma samples. Superoxide dismutase (SOD), glutathione peroxidase (GPx), catalase (CAT) activities, and also non protein thiols levels together with TEAC activity were determined on erythrocytes samples

No statistically significant changes were observed for TEAC erythrocytes, non-protein thiols, GPx and CAT activities. Significantly decreased (p<0.01) SOD activity was found in patients with androgenetic alopecia. For plasma samples decreased TEAC activity (p<0.001), increased MDA levels (p<0.001) and no change in total thiols concentration were found in patients when compared with the controls.

**Discussions:**Decreased total antioxidant activity and increased MDA levels found in plasma samples of patients with androgenetic alopecia are indicators of oxidative stress presence in these patients. Significantly decreased SOD activity but no change in catalase, glutathione peroxidase, non protein thiols level and total antioxidant activity in erythrocytes are elements which suggest the presence of a compensatory mechanism for SOD dysfunction in red blood cells of patients with androgenetic alopecia.

Abbreviations: AAG = androgenetic alopecia, MDA = malondialdehyde, SOD = superoxide dismutase, CAT = catalase, GPx = glutathione peroxidase, GSH = glutathione, GST = glutathione transferase, SH = thiols, TEAC = trolox equivalent antioxidant capacity, ABTS = 2,2’-azino-bis (3-ethylbenzothiazoline-6-sulfonic acid), CDNB = 1-chloro-2,4-dinitrobenzene

## Introduction

Androgenetic alopecia (AGA) is a non-scarring disease with a progressive thinning of the scalp hair that follows a characteristic pattern [**1**]. The pathogenesis of androgenetic alopecia involves both genetic and hormonal (androgens) factors. The hair follicles are genetically targeted for androgen stimulation leading to hair follicles miniaturization and replacement of large, pigmented hairs (terminal hairs) with shorter, thinner, depigmented hairs (vellus hairs) in affected areas [**2**]. Environmental factors (the nutritional influences, metabolic syndrome, smoking and UV radiation), also play a role in the pathogenesis of AGA [**3**,**4**]. Recent histological studies illustrated perifollicular inflammation in the upper third of the hair follicles, suggesting that inflammation plays a pathogenic role in AGA, although clinically, AGA is considered a non-inflammatory disease [**5**,**6**]. Oxidative stress and inflammation are closely linked in biological systems [**7**] so, there is also evidence of oxidative stress presence in dermal papilla cells of patients with androgenetic alopecia [**8**].

As far as we know, there is no literature report investigating the oxidative stress parameters in the blood of the patients with androgenetic alopecia. Therefore, we have considered it of interest to study the antioxidant systems in erythrocytes and plasma of these patients.

## Methods

The study was approved by the Ethics Committee of Elias Hospital in Bucharest, Romania. Prior to the initiation of the study, each patient was informed about the objectives of the study and signed an informed consent. Patients were selected from Elias Dermatology Hospital and Dermatology Outpatient Clinics, between October 2014 and May 2015. The diagnosis of androgenetic alopecia was based on the following considerations: pattern of increased hair thinning on the fronto-temporal area and vertex in men and on the frontal/ parietal scalp with retention of frontal hairline in women, family history of androgenetic alopecia, the presence of miniaturized hairs and diversity of hair diameter on dermoscopic examination. The exclusion criteria included the presence of concomitant inflammatory disease (infections, autoimmune disorders), liver and kidney diseases, thyroid disease, neoplastic diseases, recent major surgical procedures, immune-compromised patients, and diabetes mellitus. Patients who were smoking were also excluded. Patients with other dermatological conditions known to cause diffuse hair loss such as psoriasis, seborrheic dermatitis, and allergic contact dermatitis were not included. Patients exposed to medication with the potential of causing alopecia and patients who used any treatment with effects on hair growth including vitamins, anti-inflammatory drugs, topical corticosteroids, and shampoos, in the last 3 months were excluded.

The following routine laboratory tests were performed: complete blood count, liver enzymes, glucose, urea, creatinine, iron, albumin, uric acid, and total protein. Patients with abnormal laboratory findings were excluded.

Blood samples were collected after overnight fasting into lithium heparin containing tubes. Erythrocytes were separated from plasma by centrifugation and samples were kept at –8°C until analyzed. Superoxide dismutase, catalase, glutathione peroxidase, glutathione transferase activities, non-protein thiols (mainly glutathione), and Trolox Equivalent Antioxidant Activity (TEAC) were determined on erythrocytes samples. The total thiols and malondialdehyde were assayed as a measure of lipid peroxidation on TEAC plasma samples.

Total antioxidant activity (TEAC) – plasma and erythrocyte

The total antioxidant activity was determined based on the 6-hydroxy-2,5,7,8 tetramethylchroman-2 carboxylic acid (Trolox, Sigma Aldrich Chemie, Munich, Germany) equivalent antioxidant capacity assay (TEAC) developed by Miller [**9**] with modifications [**10**]. The TEAC assay measures the relative abilities of antioxidants to scavenge the 2,2′-azino-bis (3-ethylbenzothiazoline-6-sulfonic acid) (ABTS) radical cation (ABTS*+), compared with the antioxidant potency of standard amounts of Trolox, the water-soluble vitamin E analogue. The ABTS radical was generated from the interaction between ABTS and potassium persulfate. Plasma and erythrocytes samples were mixed with ABTS*+ and incubated for 1 minute at 30°C. The optical density (absorbance) was read at 734 nm against 5 mM phosphate buffered saline (pH 7.4). The percentage inhibition of absorbance, which is directly proportional to the antioxidant activity of the sample, was calculated. The assay was calibrated against a calibration curve with Trolox as the standard. TEAC was expressed as millimoles per liter of Trolox for plasma and millimoles Trolox per g haemoglobin for erythrocytes.

SOD activity

The SOD (EC. 1.15.1.1) activity was determined as described by Marklund [**11**], CuZnSOD from erythrocytes was extracted with an extraction reagent containing methanol: chloroform 62.7:37.5 (v/ v) stored at 2–8°C. After the addition of the extraction reagent, the mixture was briefly vortexed and centrifuged for 5 minutes at 3000 g and 4°C. The upper aqueous layer containing the enzyme was sampled. The rate of autoxidation of pyrogallol in the reaction buffer (TRIS-cacodylic acid 50 mM, pH = 8.2, containing 1 mM diethylene triamine pentaacetic acid DTPA) – with and without enzyme – was taken from the increase in absorbance at 420 nm. A unit of the enzyme is generally defined as the amount of enzyme that inhibits the reaction by 50%. Results are corrected for the dilution and expressed relative to the Hb content.

Erythrocyte CAT activity

CAT (EC. 1.11.1.6) activity was measured by using the method described by Aebi [**12**]. The erythrocyte lysate was diluted in 0.05 M potassium phosphate buffer (pH=7), and the reaction was started by adding 10 mM hydrogen peroxide. The decrease in absorbance at 240 nm was measured for 30 seconds. Enzyme activity was calculated as a function of the rate constant of the first order reaction (k), and was expressed as k per gram of Hb.

GPx Activity

The GPx (EC. 1.11.1.9) activity was indirectly measured by oxidation of NADPH to NADP+. 12 UI GSH reductase, 24 μM GSH and NADPH in 0,5 M TRIS-HCl buffer (pH 7.6) were added on the haemolysed diluted erythrocytes. The enzymatic reaction was initiated by adding tert butyl as a substrate. We followed the conversion of NADPH to NADP+ by a continuous recording of the decrease in the absorbance at 340nm for 3 min. GPx activity was expressed as U/ g Hb [**13**].

Non-protein thiols (non-protein SH erythrocytes)

The non-protein SH erythrocyte was determined on the erythrocyte lysate after the precipitation of proteins by using a precipitating solution containing metaphosphoric acid, disodium ethylenediaminetetraacetate, and sodium chloride. The mixture was allowed to stand for 5 minutes and then it was filtered. 0,3 M sodium phosphate solution and Ellman reagent were then added to the filtrate and the absorbance was read at 412 nm [**14**].

Results were calculated by using a calibration curve with GSH as a standard and were expressed relative to the haemoglobin content.

Glutathione transferase activity (GST)

The GST activity was spectrophotometrically determined at 340nm by measuring the formation of the conjugate of glutathione (GSH) and 1-chloro-2,4-dinitrobenzene (CDNB).

The assay system consisted of CDNB prepared in absolute ethanol and hemolysate from the sample in 0,1 M potassium phosphate buffer with a 6,25 pH to which GSH was added to initiate the reaction. The addition of GSH was done 3 minutes after the addition of CDNB. The formation of the S-conjugate was followed by the measurement of the absorbance at 340 nm. The blanks obtained without the hemolysates were subtracted from each assay value. One unit of enzyme activity was defined as the amount of enzyme that catalyzed the formation of 1uM of the S-conjugate per minute under assay conditions. Calculations were done by using the molar extinction coefficient of 9,6mM l -1 cm -1 and taking into account the dilutions. Results were expressed relative to the haemoglobin content [**15**].

Total plasma thiols assay

For the estimation of total -SH groups, content aliquots of plasma were mixed with phosphate buffer (pH = 8) and 10% sodium dodecyl sulphate. Then Ellman reagent was added and samples were incubated at 37°C for one hour. The absorbance was read at 412nm against a blank reagent. Results were calculated by using a calibration curve with GSH [**16**].

Thiobarbituric acid test (MDA)

Aliquots of plasma were mixed with butylated hydroxytoluene (BHT), 0,67% thiobarbituric acid and 20% trichloroacetic acid followed by incubation at 100°C for 1 hour; the reaction was stopped by cooling the samples with tap water. The pink MDA-TBA adduct was extracted in n-butanol and the absorbance of the organic layer was read at 532 nm after centrifugation. The concentration of lipid peroxidation products was calculated by using a calibration curve prepared with 1,1,3,3-tetramethoxypropane as the standard and expressed as nmol MDA equiv./ g protein. The absorbance measured this way can come from all pre-existing MDA, protein-bound MDA and lipid peroxides, as well as any other substances that give rise to MDA or TBARS in the hot acid. BHT was added to the sample prior to the assay, to ensure that no lipid oxidation occurred during the assay procedure [**17**].

### Statistical analysis

Data were expressed as mean ± SEM. Comparisons between groups were performed by student t test (parametric or non-parametric as needed). The relationships between different parameters were assessed by Pearson’s test and GraphPadInStat software (GraphPad Software Inc.La Jolla, United States). A p-value < 0.05 was considered to be statistically significant.

## Results

Twenty-seven patients with AGA (20 females and 7 males), aged 35,40 ± 9,35 years (mean + SD) were enrolled in the study. The mean duration of the disease was of 4,61± 4,04 years. Twenty-five control subjects were included in this study (24 females and 1 man), aged 32 ± 8,78 years (mean + SD). The controls were selected volunteers from Elias Dermatology Hospital staff.

The biochemistry assays results are presented in **Table 1** as average + standard error of the mean values. TEAC Erythrocytes was not significantly different between groups. Superoxide dismutase activity showed a statistically significant decrease for subjects with androgenetic alopecia (p<0.01) from 546.85 + 21.99 U/ g Hb. in the control group to 441.35 + 26.83 U/ g Hb. in the androgenetic alopecia group.

**Table 1:**
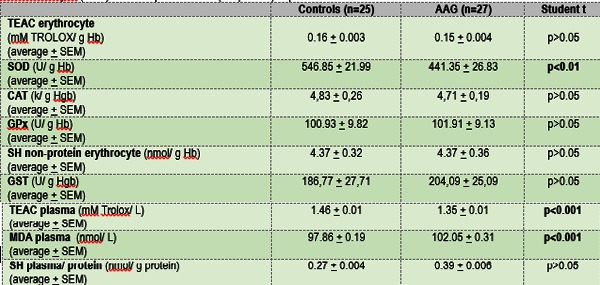
**Table 1**. Antioxidant and oxidative stress parameters in patients with androgenetic alopecia. Total antioxidant capacity in plasma and erythrocyte (TEAC erythrocyte, TEAC plasma), superoxide dismutase (SOD), non-protein erythrocytes thiol (SH non-protein erythrocyte), catalase (CAT), glutathione peroxidase (GPx), glutathione transferase (GST), malondialdehyde (MDA) and total plasma thiols (SH plasma) expressed as media + standard error of the mean with ANOVA results

Catalase activity values were very similar for both groups (4.83 k/ g Hb Ctrl and 4.71 k/ g Hb AAG) and no statistical difference could be found. The same situation was registered for glutathione peroxidase activity, non-protein erythrocyte thiols and glutathione transferase activity, where p>0.05 was found for the two studied groups.

TEAC and MDA plasma values showed very significant statistical differenc es (P <0.001) between groups, plasma TEAC for patients with androgenetic alopecia being significantly decreased when compared with the controls, while the MDA levels were significantly increased in patients compared with the controls.

Plasma thiols levels were not significantly changed in the patients group compared with the control group.

A statistically significant correlation was found between the SOD erythrocytes activity and plasma TEAC values, (r) = 0.4204, p=0.0364 (two tailed) in the control group. TEAC and SOD erythrocytes activity were significantly correlated: (r) = 0.6590, p= 0.0002 in the patients group.

## Discussion

The presence of oxidative stress in dermal papilla of the patients with androgenetic alopecia was recently confirmed [8]. There are also several literature reports that can link androgenetic alopecia to increased oxidative stress - androgenetic alopecia was associated with increased risk factors for cardiovascular disease and with increased presence of inflammation [**6**,**18**,**19**,**20**]. Oxidative stress generated by an increased availability of substrates prone to oxidative damage may be a trigger for the inflammatory response in cardiovascular disease [**21**]. The disturbed metabolism of copper and zinc observed in the serum, urine and hair of the patients with androgenetic alopecia [**22**] might also increase oxidative stress [**23**].

The increased MDA level, a marker of lipid peroxidation together with a decreased TEAC observed in our study, in the plasma of the patients with androgenetic alopecia suggested that an increased oxidative stress was present in these patients.

Erythrocytes are among the cells chronically exposed to free radicals damage due to increased oxygen concentration and also increased iron levels in hemoglobin. Therefore, erythrocytes were equipped with a strong antioxidant defense mechanism including CuZn superoxide dismutase, an enzyme transforming superoxide radical in hydrogen peroxide, further decomposed in water and oxygen by catalase. Using glutathione as a cofactor, glutathione peroxidase transforms peroxides into less toxic products and is also an important part of erythrocytes antioxidant defense enzymatic system. Interestingly, these enzymes themselves may be inactivated by free radicals [**24**]. Our study found a decreased CuZn SOD activity in the erythrocytes of the patients with androgenetic alopecia but no other significant difference in the activity of the other antioxidant systems – catalase and glutathione peroxidase activities was noticed and also no change in non-protein thiols levels was observed. Moreover, the TEAC total antioxidant activity, a measure of synergetic actions of all antioxidants present in a biological system, is not significantly changed between patients and controls in erythrocytes. These results suggested only a disturbance in the SOD activity and also that the other antioxidant systems present in the erythrocytes were able to compensate this deficit in our patients. Thus, we could not rule out a disturbance in the copper metabolism observed by others [**22**] in these patients, as a cause of decreased SOD activity observed by us. However, there is no literature report considering copper levels determinations in erythrocytes of patients with androgenetic alopecia and we did not determine it in our study.

## Conclusion

The decreased total antioxidant activity and the increased MDA levels found in the plasma samples of the patients with androgenetic alopecia are indicators of oxidative stress presence in these patients. Significantly decreased SOD activity but no change in catalase, glutathione peroxidase, non-protein thiols level and total antioxidant activity in erythrocytes suggest the presence of a compensatory mechanism for SOD dysfunction in red blood cells of patients with androgenetic alopecia.

**Acknowledgement:**This paper is supported by the Sectorial Operational Programme Human Resources Development (SOP HRD), financed from the European Social Fund and by the Romanian Government under the contract number POSDRU/159/1.5/S/137390.

**Disclosures:**None

## References

[1] Torres F (2015). Androgenetic, diffuse and senescent alopecia in men: practical evaluation and management. Curr. Probl. Dermatol..

[2] Hoffmann R (2002). Male androgenetic alopecia. Clin. Exp. Dermatol..

[3] Rushton DH (2002). Nutritional factors and hair loss. Clin. Exp. Dermatol..

[4] Trueb RM (2003). Is androgenetic alopecia a photoaggravated dermatosis?. Dermatology..

[5] Aslani FS, Dastgheib L, Banihashemi BM (2009). Hair counts in scalp biopsy of males and females with androgenetic alopecia compared with normal subjects. J. Cutan. Pathol..

[6] Magro CM, Rossi A, Poe J, Manhas-Bhutani S, Sadick N (2011). The role of inflammation and immunity in the pathogenesis of androgenetic alopecia. J. Drugs Dermatol..

[7] Wadley AJ, Veldhuijzen van Zanten JJCS, Aldred S (2013). The interactions of oxidative stress and inflammation with vascular dysfunction in ageing: the vascular health triad.. Dordr.

[8] Upton JH, Hannen RF, Bahta AW, Farjo N, Farjo B, Philpott MP (2015). Oxidative stress-associated senescence in dermal papilla cells of men with androgenetic alopecia. J. Invest. Dermatol..

[9] Miller NJ, Rice-Evans CA (1996). Spectrophotometric determination of antioxidant activity. Redox Rep..

[10] Re R, Pellegrini N, Proteggente A, Pannala A, Yang M, Rice-Evans C (1999). Antioxidant activity applying an improved ABTS radical cation decolorization assay. Free Radic.. Biol. Med..

[11] Marklund S, Marklund G (1974). Involvement of the superoxide anion radical in the autoxidation of pyrogallol and a convenient assay for superoxide dismutase. Eur. J. Biochem..

[12] Aebi H (1984). Oxygen Radicals in Biological Systems. Methods. Enzymol.

[13] Ursini F, Maiorino M, Brigelius-Flohe R, Aumann KD, Roveri A, Schomburg D, Flohe L (1995). Diversity of glutathione peroxidases. Methods. Enzymol.

[14] Beutler E, Duron O, Kelly BM (1963). Improved method for the determination of blood glutathione. J. Lab. Clin. Med..

[15] Habig WH, Jakoby WB (1981). Glutathione S-transferases (rat and human). Methods Enzymol..

[16] Rice-Evans C (1991). Techniques in Free Radical Research. vol. 22. Elsevier..

[17] Esterbauer H, Cheeseman KH (1990). Determination of aldehydic lipid peroxidation products: malonaldehyde and 4-hydroxynonenal. Methods Enzymol.

[18] Arias-Santiago S, Gutiérrez-Salmerón MT, Castellote-Caballero L, Buendía-Eisman A, Naranjo-Sintes R (2010). Androgenetic alopecia and cardiovascular risk factors in men and women: a comparative study. J. Am. Acad. Dermatol..

[19] Ertas R, Orscelik O, Kartal D, Dogan A, Ertas SK, Aydogdu EG, Ascioglu O, Borlu M (2015). Androgenetic alopecia as an indicator of metabolic syndrome and cardiovascular risk. Blood Press..

[20] Arias-Santiago S, Gutiérrez-Salmerón MT, Buendía-Eisman A, Girón-Prieto MS, Naranjo-Sintes R (2011). Sex hormone-binding globulin and risk of hyperglycemia in patients with androgenetic alopecia. J. Am. Acad. Dermatol..

[21] Hulsmans M, Holvoet P (2010). The vicious circle between oxidative stress and inflammation in atherosclerosis. J. Cell. Mol. Med..

[22] Ozturk P, Kurutas E, Ataseven A, Dokur N, Gumusalan Y, Gorur A, Tamer L, Inaloz S (2014). BMI and levels of zinc, copper in hair, serum and urine of Turkish male patients with androgenetic alopecia. J. Trace Elem. Med. Biol..

[23] Gaetke LM, Chow CK (2003). Copper toxicity, oxidative stress, and antioxidant nutrients. Toxicology.

[24] Pigeolet E, Corbisier P, Houbion A, Lambert D, Michiels C, Raes M, Zachary MD, Remacle J (1990). Glutathione peroxidase, superoxide dismutase, and catalase inactivation by peroxides and oxygen derived free radicals. Mech. Ageing Dev..

